# Sigmoid Schistosomiasis Granuloma Manifested as a Large Painless Supra‐Pubic Mass: A Case Report

**DOI:** 10.1002/ccr3.70681

**Published:** 2025-07-29

**Authors:** Rawa Badri, Safa Abdalrhim, Aymen Hamid, Ahmed Salah Aldeen, Ahmed Tahir, Mohamad Osama Ahmad

**Affiliations:** ^1^ Mycetoma Research Centre Khartoum Sudan; ^2^ Faculty of Medicine, University of Khartoum Khartoum Sudan; ^3^ Sudanese Medical Specialization Board Khartoum Sudan; ^4^ Faculty of Medicine, National Ribat University Khartoum Sudan; ^5^ Faculty of Medicine, University of Kassala Kassala Sudan; ^6^ Department of General Surgery New Halfa Teaching Hospital Kassala Sudan

**Keywords:** abdominal mass, granuloma, NTDs, schistosomiasis, Sudan

## Abstract

Schistosomiasis remains a prevalent parasitic disease in tropical and subtropical regions, ranking second to malaria in terms of parasitic burden. The disease manifests in two primary forms: urinary schistosomiasis, caused by Schistosoma haematobium, and intestinal schistosomiasis, associated with *S. mansoni* and *S. japonicum* . This case report describes a 12‐year‐old female from New Halfa, Sudan, who presented with a progressively enlarging lower abdominal mass. She had a prior history of *S. mansoni* infection, diagnosed and treated a year earlier. Clinical evaluation revealed a firm, mobile, non‐tender suprapubic mass, while imaging studies identified a large abdominopelvic mass with mesenteric lymphadenopathy. Surgical exploration confirmed a sigmoid colon tumor, necessitating resection and anastomosis. Histopathological analysis revealed viable Schistosoma ova with granulomatous inflammation, confirming colonic schistosomiasis. The patient recovered well postoperatively and received praziquantel therapy. This case underscores the importance of early schistosomiasis treatment to prevent severe complications, including granuloma formation and intestinal obstruction. It also highlights the diagnostic challenges of intestinal schistosomiasis, which can mimic neoplastic conditions. Enhanced screening, timely praziquantel administration, and improved public health interventions are crucial in endemic areas to mitigate disease progression and long‐term morbidity.

**Summary**

- This case highlights an unusual presentation of intestinal schistosomiasis as a granulomatous mass in the colon.
- It underscores the critical role of histopathological examination in distinguishing schistosomiasis from other conditions like malignancies.
- Early diagnosis, appropriate surgical management, and targeted treatment with praziquantel are vital for improving patient outcomes, especially in endemic areas.

## 1 Introduction

Schistosomiasis is a major parasitic disease in tropical and subtropical regions and is considered the second most common parasitic infection after malaria in these areas [1]. The disease has two clinical forms: urinary schistosomiasis, which is caused by Schistosoma haematobium, and intestinal schistosomiasis, which is caused by *S. mansoni* or Schistosoma japonicum [2]. Schistosomiasis has been reported across multiple regions of Sudan, including Wadi Halfa, the Northern State, Darfur, Kassala, Gadaref, Kordofan, Gezira, White Nile, and various parts of Khartoum. In the New Halfa locality in Kassala state, there is a notable prevalence of *S. mansoni* . Additionally, *S. mansoni* infection is more frequently seen in young children and is significantly more prevalent in females compared to males [1].

Schistosomiasis is transmitted when a person comes into contact with freshwater containing schistosoma eggs, which hatch into parasites that penetrate the skin. The parasite follows a complex life cycle, beginning with the entry of cercariae—larval forms released by freshwater snails—into the mammalian host. After penetration, these larvae develop into schistosomula and move through the bloodstream and lymphatic system, eventually reaching the lungs. They then migrate to the liver's venous system, where they mature, pair, and become capable of reproduction. To complete the cycle, the *S. mansoni* eggs traverse into the intestinal lumen and are eliminated in the host's feces, typically by the sixth week of infection [3]. Nevertheless, it is important to mention that as the eggs migrate, some may get trapped in the intestine or liver sinusoids. This can trigger an immune response involving CD4+ T cells, leading to the formation of granulomas [3].

In this case, we hypothesize that chronic *Schistosoma mansoni* infection, particularly in endemic regions with delayed or incomplete treatment, may lead to granulomatous inflammation severe enough to mimic neoplastic conditions, complicating diagnosis and management. The objectives of this case report are to emphasize the importance of early medical treatment of schistosomiasis to prevent such progression and the necessity of histopathological examination in differentiating schistosomiasis granulomas from other masses.

## 2 Case History and Examination

A 12‐year‐old female from New Halfa locality, Kassala state, presented to our surgery clinic in January 2023 with a chief complaint of a lower abdominal mass that started 8 months before her presentation. The mass was of gradual onset and progressed over time. It was painless, mobile, and did not associate with any other symptoms. The patient has a history of blood in stool, where she was diagnosed with intestinal schistosomiasis caused by *S. mansoni* and received a single dose of praziquantel one year before her presentation. There is no family history of a similar condition.

She is from a low socioeconomic status family with regular environmental exposure to unsafe water sources, as well as residing in close proximity to a water canal in a highly endemic area for schistosomiasis in Sudan.

On clinical examination, the patient looked unwell but she was not jaundiced or pale. Haemodynamically, she was stable. Her pulse rate was 100 beats per minute, her respiratory rate was 20 breaths per minute, and she was not febrile. Her abdominal examination showed a palpable, mobile, and non‐tender supra‐pubic mass that was hard in consistency with an irregular surface and measured 10 × 8 cm in diameter. Otherwise, the abdomen was soft, not distended, and no organomegaly or other masses were detected. Other systems examinations were unremarkable.

## 3 Methods (Investigations, Differential Diagnosis, and Management)

Laboratory investigations revealed a hemoglobin level of 9.9 g/dL, with a normal platelet count of 290 × 10^9^/L, a total white blood cell count of 7.7 × 10^9^/L, and an erythrocyte sedimentation rate (ESR) of 95 mm/h.

The patient had several imaging studies including an abdominal Ultrasound scan and a Computed Tomography (CT) scan. The abdominal Ultrasound showed a large solid homogeneous mass with smooth margins compressing the jejunum and measuring 7.3 × 4.5 × 3.6 cm.

**FIGURE 1:**
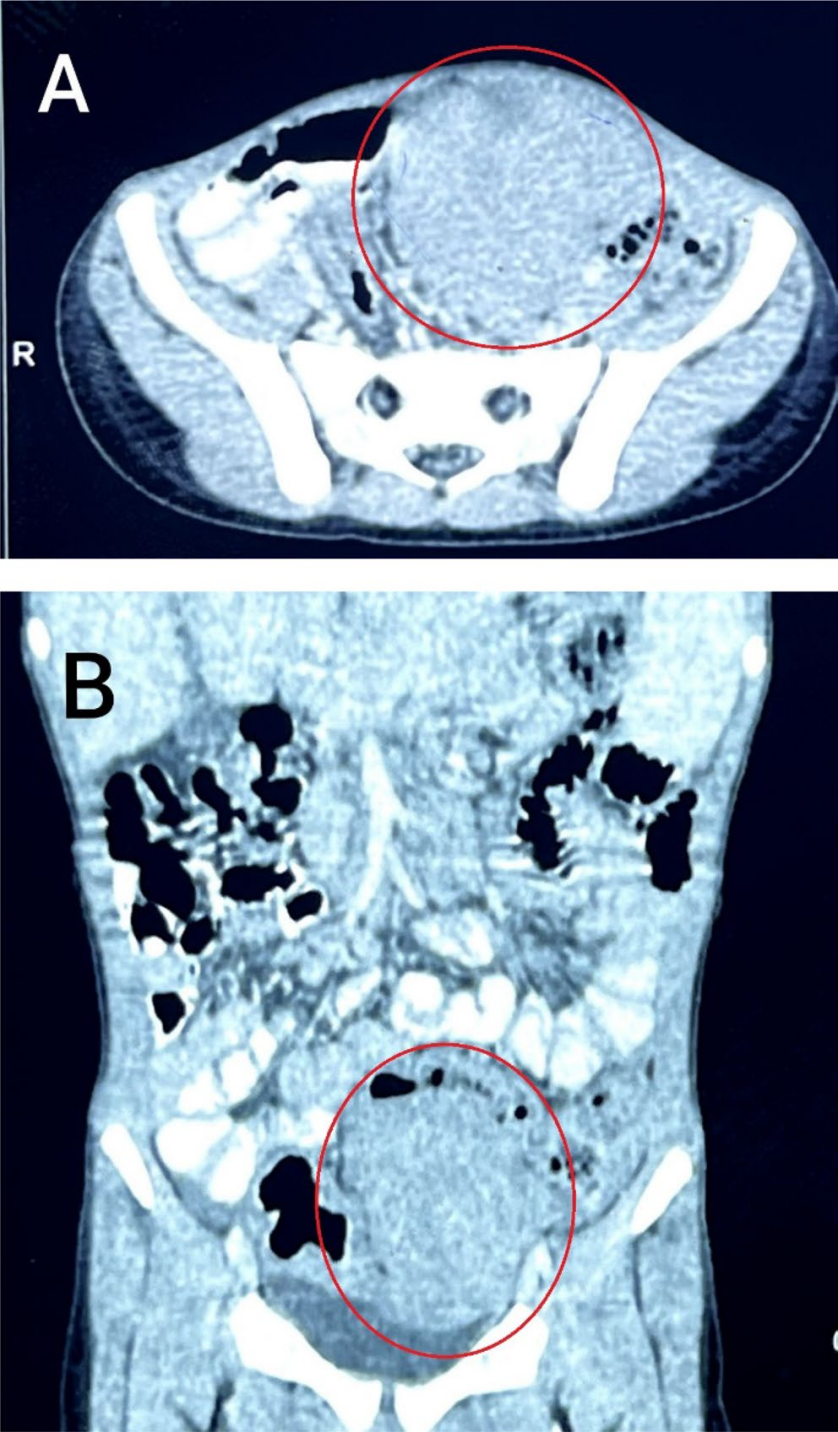
Abdominal computed tomography (CT) scan showing (A) an axial section of the abdominopelvic mass narrowing the sigmoid colon lumen and (B) a coronal section of the mass.

The CT scan revealed an abdominopelvic solid mass, mainly to the left measuring 7.5 × 6.6 × 6.8 cm. There were multiple mesenteric lymph nodes with an average size of 15 mm (Figure 1). The CT scan also showed a normal liver size and tissue density with no focal or enhancing lesions and normal intra‐ and extra‐hepatic biliary radicles.

**FIGURE 2:**
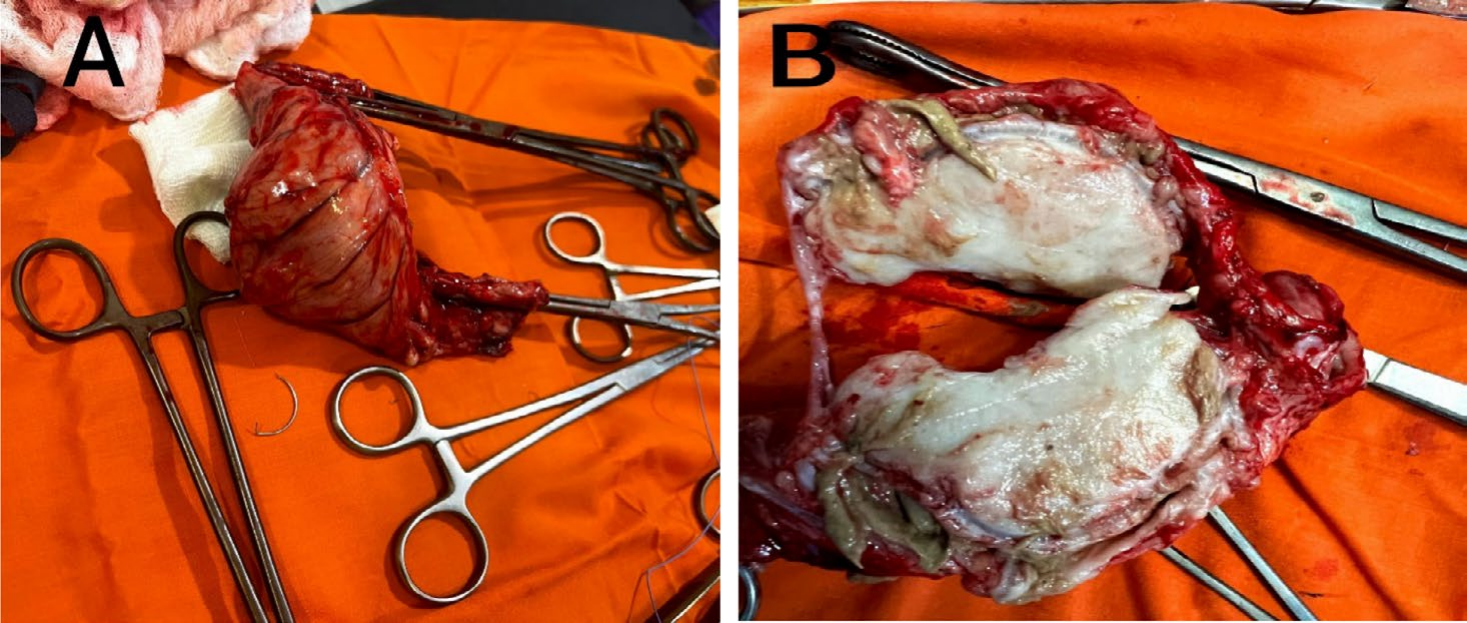
Photograph of the mass showing (A) the resected mass with part of the sigmoid colon and (B) a sagittal section of the resected mass.

Differential diagnoses of lymphoma or leiomyosarcoma were considered. The patient was admitted to the hospital on the 30th of January 2023 and prepared for an exploratory laparotomy. Intraoperatively, there was a large sigmoid tumor measuring about 12 × 6 cm (Figure 2). Also, there were multiple enlarged mesenteric lymph nodes and multiple hepatic nodules. Resection and anastomosis of the sigmoid colon with safety margins about 5 cm above and below the tumor were done. The resected mass and mesenteric lymph node biopsies were sent for histopathological examination.

## 4 Conclusion and Results

**FIGURE 3:**
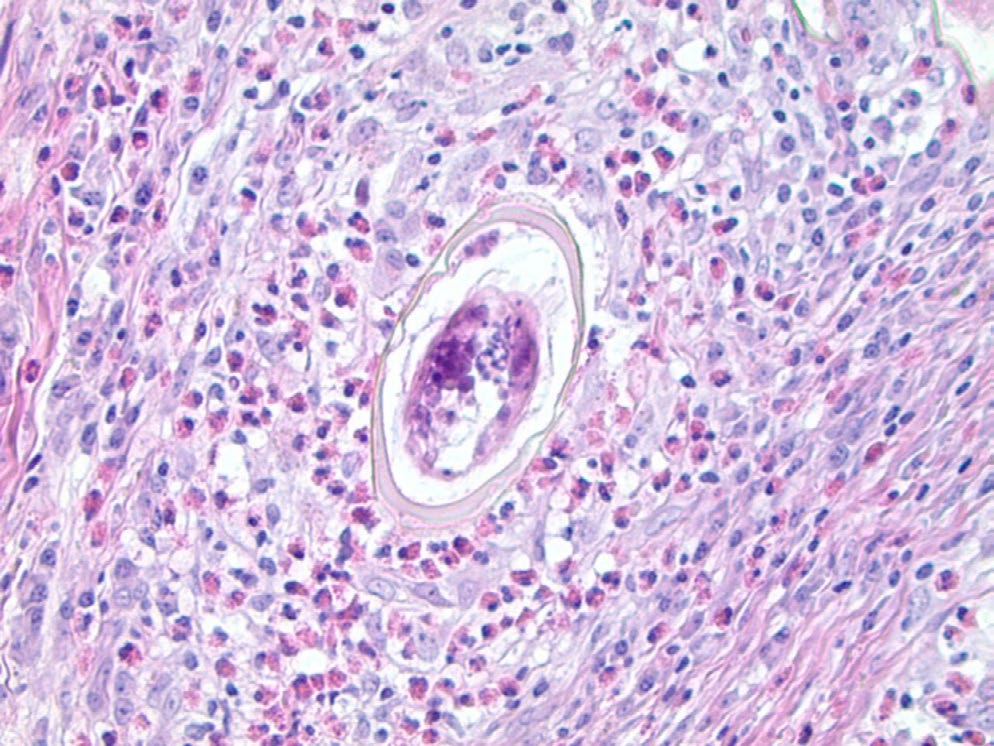
Hematoxylin‐and‐eosin‐stained section showing *S. mansoni* egg in the center of a granuloma [4].

The patient was discharged on Day 4 from the surgical ward in good condition. A histopathological study of the resected segment which contains the sigmoid mass and mesenteric lymph node was done and it showed viable Schistosome ova associated with histiocytic and giant cell granulomas rich in eosinophils in the submucosa and serosa of the sigmoid colon as well as the mesenteric lymph node (Figure 3). No evidence of malignancy is noted in sections of adequate biopsy examined.

Following the surgical intervention, the patient was referred to the Pediatrics department where she received a singular oral dosage of praziquantel of 40 mg/kg as a treatment for schistosomiasis and continued to follow up with them.

Two weeks following the surgical operation, the patient presented to the refer clinic for follow‐up. She was well, with no unpleasant symptoms, a normal abdominal examination, and good surgical wound healing.

## 5 Case Discussion

People with colonic schistosomiasis mostly experience nonspecific symptoms including abdominal pain, constipation, diarrhea, and rectal bleeding. In addition, severe pathological disorders associated with schistosomiasis have been reported, such as obstruction due to inflammatory masses, ischemic colitis, intussusception, acute appendicitis, and colon cancer [5]. The patient presented with a progressively enlarging suprapubic mass, a rare but recognized complication of chronic *Schistosoma mansoni* infection. While schistosomiasis typically causes nonspecific gastrointestinal symptoms, granulomatous inflammation can lead to significant mass formation, as seen in this case. Laboratory findings showed mild anemia (Hb 9.9 g/dL) and an elevated ESR (95 mm/h), both indicative of chronic inflammation but not specific for schistosomiasis. Imaging studies revealed a large, well‐defined solid mass with mesenteric lymphadenopathy, initially raising suspicion of lymphoma or leiomyosarcoma. However, the absence of features such as necrosis, invasive characteristics, or distant metastases lowered the likelihood of malignancy. Histopathological analysis confirmed the diagnosis by identifying viable *Schistosoma* ova surrounded by eosinophil‐rich granulomas and fibrosis. This characteristic inflammatory response is essential for distinguishing schistosomiasis‐related masses from malignancies. This case underscores the importance of integrating clinical, laboratory, and imaging findings to differentiate parasitic infections from malignancies, especially in endemic areas [6].

There have been several documented cases of intestinal polyps induced by schistosomiasis. One of the cases involved non‐necrotizing colonic schistosomiasis polyps, presented with abdominal pain and diarrhea [7]. Another case presented with a prolapsed rectal polyp accompanied by rectal bleeding and constipation, while a third case experienced intermittent abdominal pain and constipation [8]. In all cases, colonoscopy was done, and it revealed a sessile polyp measuring approximately 5–10 mm in size in the sigmoid colon, a large mobile polyp with a stalk measuring 2 × 2.5 cm in the rectum, and nonspecific erythema in the sigmoid colon, respectively. All cases were treated by snare cautery polypectomy. Histopathological examination revealed the presence of schistosome eggs accompanied by an inflammatory reaction in all reported cases [7, 8]. Nevertheless, in our reported case, the size of the mass was large, measuring 7.5 × 6.6 × 6.8 cm, necessitating the performance of exploratory laparotomy for complete resection.

Conversely, another case presented with severe symptoms, including intestinal obstruction and the presence of a lower abdominal mass [9]. A CT scan of the abdomen revealed mild hepato‐splenomegaly, lymph node enlargement, and a significantly thickened wall in the rectosigmoid and descending colon [9]. Initially, the patient underwent laparotomy; however, the condition was determined to be inoperable. Subsequently, a sigmoidectomy was performed, followed by the creation of a colostomy. After three months, an end‐to‐end anastomosis was performed and closure of the colostomy was done [9].

Other presentations include gastrointestinal symptoms that mimic cancer as reported in a case presented with a huge colonic granuloma with a chief complaint of abdominal pain, vomiting, right iliac fossa mass, anorexia, and weight loss. Abdominal U/S and CT scans were done to exclude the probability of cancer, and then exploratory laparotomy with complete resection was done [10]. However, in our particular case, the patient presented with a painless, progressively enlarging supra‐pubic mass without any signs of obstruction or other associated symptoms, posing a challenge in reaching a diagnosis.

Intestinal masses due to schistosomiasis granuloma are extremely rare. The patient's young age is a crucial factor in this case. Schistosomiasis predominantly affects children and adolescents in endemic regions due to frequent contact with contaminated water sources [2]. Younger patients often present with nonspecific symptoms, which can delay diagnosis and lead to complications, such as granulomatous masses mimicking neoplasms. Additionally, chronic inflammation in growing tissues may have influenced the formation and progression of the lesion.

This case supports the hypothesis that prolonged *S. mansoni* infection can lead to significant granulomatous masses, presenting as an abdominal tumor‐like lesion. The absence of early symptoms and the slow progression of such masses may delay clinical suspicion, leading to unnecessary invasive procedures. This highlights the need for early diagnosis, prompt treatment with praziquantel, and regular follow‐up in endemic regions to prevent severe complications. In this patient, surgical resection of the mass is the treatment of choice; thus, laparotomy with resection and anastomosis of the sigmoid colon was done. The young age of the patient and her good general health state are important factors for the good prognosis of her condition postoperatively. Early recognition and intervention are essential to prevent severe morbidity in pediatric and adolescent populations. This case reinforces the need for targeted screening and health education in school‐aged children living in endemic areas.

In conclusion, early diagnosis and a multidisciplinary approach are essential for managing sigmoid schistosomiasis granuloma and preventing misdiagnosis. Histopathological evaluation remains crucial for distinguishing it from other colonic masses. Future research should focus on advanced diagnostic tools and novel therapeutic strategies to enhance early detection and treatment outcomes in endemic regions.

## Author Contributions

**Rawa Badri:** conceptualization, data curation, writing – original draft, writing – review and editing. **Safa Abdalrhim:** conceptualization, data curation, writing – original draft, writing – review and editing. **Aymen Hamid:** data curation, writing – original draft. **Ahmed Salah Aldeen:** data curation, writing – original draft. **Ahmed Tahir:** conceptualization, data curation, supervision, writing – original draft, writing – review and editing. **Mohamad Osama Ahmad:** conceptualization, data curation, supervision, writing – original draft, writing – review and editing.

## Consent

Written informed consent was obtained from the patient to publish this report in accordance with the journal's patient consent policy.

## Conflicts of Interest

The authors declare no conflicts of interest.

## Data Availability

The authors have nothing to report.
